# Foreword: Preventing COVID-19 Among the Immunocompromised Population

**DOI:** 10.1093/infdis/jiad106

**Published:** 2023-08-04

**Authors:** Michelle Willicombe

**Affiliations:** Department of Immunology and Inflammation, Imperial College London, London, UK

**Keywords:** COVID-19, immunocompromised individuals, mRNA-based vaccines

## Abstract

The COVID-19 pandemic represented 1 of the more significant and unique public health challenges facing the global community, particularly afflicting those with compromised immune systems. These immunocompromised individuals were readily recognized as a group at high risk of infection by SARS-CoV-2 and the associated severe outcomes of COVID-19. Although preventive strategies such as vaccination are important, initial clinical vaccine trials did not enroll immunocompromised individuals; in-depth evaluations of the safety, immunogenicity, and real-world outcomes associated with these vaccines were conducted in this population thereafter. As immunogenicity data of COVID-19 vaccination among this disparate group of individuals emerged, vaccination strategies were adapted to address outstanding challenges and further protect the entirety of this population. This 8-part journal supplement characterizes in-depth the mRNA-based COVID-19 vaccination strategies across the spectrum of immunocompromised individuals, focusing on the ongoing approaches to challenges facing this group as the pandemic continues to evolve.

The COVID-19 pandemic has represented one of the more significant and unique public health challenges facing the global community. Ensuring that measures were rapidly and effectively in place to protect the most vulnerable populations, such as individuals with compromised immune systems, was a critical factor within the pandemic response. Immunocompromised individuals were readily recognized as a group at high risk of infection by the causative pathogen, SARS-CoV-2, and the associated severe outcomes of disease. Preventive strategies such as vaccination were thus important; however, there were significant hurdles in protecting the immunocompromised populations through vaccination in terms of both safety and effectiveness. At the beginning of the COVID-19 pandemic, vaccines based on mRNA technology were developed at an unprecedented pace and were initially authorized for use beginning in December 2020, including for use in immunocompromised persons. While initial clinical trials did not enroll these more susceptible populations, observational and real-world studies have conducted in-depth evaluations of the safety, immunogenicity, and real-world outcomes associated with these vaccines in different groups of immunocompromised populations. As information on the immunogenicity of COVID-19 vaccination among this disparate group of individuals emerged, vaccination strategies have similarly adapted to address outstanding challenges and further ensure that the entirety of this population remains protected. This journal supplement provides an in-depth characterization of mRNA-based COVID-19 vaccination across the spectrum of immunocompromised individuals, focusing on the insight gained and the ongoing approaches to challenges facing this group as the pandemic continues to evolve and potentially becomes endemic.

The article by Antinori and Bausch-Jurken [1] provides a general overview on the immunocompromised population, emphasizing the lack of homogeneity across different patient groups, both in respect to COVID-19 burden and immune responses to mRNA-based vaccination. The authors broadly summarize the factors influencing immune responses to mRNA-based COVID-19 vaccines, including patient characteristics such as the type of immunosuppressive therapy received or the degree of immunosuppression, and vaccine-specific factors related to the vaccination regimen. Overall, the article reviews the opportunities and challenges of vaccinating this disparate group of individuals, emphasizing the importance of this knowledge for guiding effective healthcare in this population.

The article by Finckh and colleagues [2] focuses on patients with immune-mediated inflammatory diseases (IMIDs), a variable group of diseases with a common etiology of immune dysregulation. The authors describe the increased susceptibility to SARS-CoV-2 infection among this population and its associated severe outcomes, while also highlighting that the primary contributor to these poor outcomes is likely the immunosuppressive medications prescribed for patients with IMIDs as opposed to the IMIDs themselves. Likewise, these therapies can also blunt immune responses to mRNA-based COVID-19 vaccination, with treatments such as rituximab (anti-CD20 B-cell–depleting therapy) and abatacept (costimulation blockade) associated with the lowest antibody responses after a 2-dose series. The authors describe how vaccination strategies have adapted to enhance vaccine-elicited immunogenicity, including administration of multiple doses. Overall, despite the variable and lowered immune responses in these patients relative to the general population, COVID-19 vaccination remains important, as it is still expected to provide protection against severe disease outcomes.

The article by Dr Paris [3] collates information on disease burden and mRNA-based COVID-19 vaccination for patients with inborn errors of immunity—rare disorders wherein at least 1 immune system component is compromised. The etiology and characteristics of disease among this population are quite distinctive between patients, which has added complications to deciphering the burden of COVID-19 and the impact of vaccination among this group. Paris describes how the majority of the available literature for this population is limited to those with combined immunodeficiencies (CIDs) or predominantly antibody deficiencies (PADs), with a relative dearth of information for the remaining patient groups. Patients with CIDs or PADs have a risk for severe outcomes stemming from SARS-CoV-2 infection, which supports the importance of vaccination in these patients. However, overall evidence on immune responses to vaccination are lacking, although findings from some studies suggest that vaccination can provide some level of protection against severe disease in patients with CIDs or PADs. The article emphasizes the need for additional studies evaluating the immunogenicity of mRNA-based COVID-19 vaccines in patients with primary immunodeficiencies.

The article by Dr Subramanian [4] highlights findings among solid organ transplant recipients (SOTRs), a population receiving immunosuppressive therapies to prevent organ rejection after transplant. Dr Subramanian reviews the influence of these treatments on responses to mRNA-based COVID-19 vaccination, describing how reduced immune responses in SOTRs are associated with the use of certain immunosuppressive therapies. The article also describes the evolution of vaccination approaches to bolster immune responses among this population, particularly among patients who were initially nonresponsive to a 2-dose series. Overall, additional information on the characteristics of mRNA-based COVID-19 vaccination remains warranted in this group, which can help to further determine the needed measures to ensure their protection.

The article by Rouphael and Bausch-Jurken [5] reviews the burden of COVID-19 and vaccination strategies in patients receiving dialysis; these patients remain at elevated risk for infection due to a multitude of factors that impair the immune system. The authors provide an overview of the known literature on immune responses to mRNA-based COVID-19 vaccines in these patients, with a focus on an extended 3-dose primary regimen. The third primary dose provides much-needed boosting to the low and waning responses observed in this population following the initial 2-dose primary regimen. Real-world outcomes of vaccination in this population are also explored, providing an overview of the beneficial impact of mRNA-based COVID-19 vaccination among dialysis recipients.

The article by Hall and Teh [6] focuses on patients with cancer and patients receiving hematopoietic stem cell transplant or chimeric antigen receptor T-cell therapy (CAR-T). The authors review the burden of COVID-19 among this population, highlighting the poor outcomes for patients with cancer, particularly those with hematologic malignancies who are treated with CAR-T therapy. Vaccination is thus important, particularly in the context of additional vaccine doses to bolster immune responses. Notably, the authors also describe the key finding that despite low antibody responses, COVID-19 vaccination can still be beneficial in this population as cellular immunity responses to COVID-19 vaccination can occur among patients with cancer. Overall, additional evaluations of the characteristics of mRNA-based COVID-19 vaccination in this population are warranted, particularly as the pandemic continues to evolve.

In the concluding article of the supplement, Bonanni and colleagues [7] summarize the key findings of the previous articles describing COVID-19 vaccination in heterogeneous immunocompromised populations, as well as discuss the current challenges to determining the most effective vaccination strategies for these at-risk individuals. The authors consider how to approach vaccinating this population as the COVID-19 pandemic progresses and potentially transitions to an endemic stage. These challenges include the need to better define and communicate immunization schedules for the heterogenous groups of immunocompromised patients, particularly regarding language surrounding primary and additional/booster vaccination. The need for tailoring patient care is also discussed, as well as minimizing vaccine hesitancy. Finally, challenges surrounding vaccination in relation to an evolving COVID-19 pandemic with emerging variants of concern are considered, while pragmatic issues related to integrating COVID-19 vaccination into immunization schedules, as well as vaccine access and procurement, are also examined.

As a compilation, this supplement intends to serve as a detailed reference for healthcare professionals and policymakers who continue to assess and care for this population in the midst of the ongoing and evolving COVID-19 pandemic. Readers of these articles will be well-informed on the characteristics of mRNA COVID-19 vaccination across the spectrum of immunocompromised patients to aid in addressing challenges and sustaining momentum for protective efforts in these groups.

## Notes

***Acknowledgments***. Medical writing and editorial assistance were provided by Emily Stackpole, PhD, and Louansha Nandlal, PhD, of MEDiSTRAVA, in accordance with Good Publication Practice (GPP 2022) guidelines, funded by Moderna, Inc., and under the direction of the author.

***Supplement sponsorship.*** This article appears as part of the supplement “COVID-19 Vaccination in the Immunocompromised Population,” sponsored by Moderna, Inc.
